# Surgical Site Infections in Elective and Emergency Abdominal Surgeries: A Prospective Observational Study About Incidence, Risk Factors, Pathogens, and Antibiotic Sensitivity at a Government Tertiary Care Teaching Hospital in India

**DOI:** 10.7759/cureus.48071

**Published:** 2023-10-31

**Authors:** Himanshu Jatoliya, Rajendra K Pipal, Dharmendra K Pipal, Prakash Biswas, Vibha Rani Pipal, Seema Yadav, Bhavna Verma, Vikram Vardhan

**Affiliations:** 1 General Surgery, Government Medical College, Sirohi, IND; 2 Orthopedics, Geetanjali Medical College, Udaipur, IND; 3 General, Colorectal, and Minimal Access Surgery, All India Institute of Medical Sciences, Gorakhpur, IND; 4 General Surgery, All India Institute of Medical Sciences, Gorakhpur, IND; 5 Obstetrics and Gynaecology, All India Institute of Medical Sciences, Gorakhpur, IND; 6 Anesthesia, Rajmata Vijaya Raje Scindia Medical College, Bhilwara, IND; 7 Anti-retroviral Therapy (ART) Centre, Government Medical College, Sirohi, IND; 8 Anesthesiology, Pain Medicine, and Critical Care, All India Institute of Medical Sciences, Gorakhpur, IND

**Keywords:** diabetes mellitus, microbial pathogens, surgical site infection, risk factors, nosocomial infection, infection control, antimicrobial sensitivity, antibiotic resistance, antibiotic prophylaxis, abdominal surgery

## Abstract

Background

Surgical site infections (SSIs), the third most common nosocomial infection, endanger hospitals and patients. SSIs must be monitored continuously. This present study examined SSI incidence, risk factors, pathogens, and antibiotic sensitivity in emergency and elective or planned abdominal surgeries.

Methods

The Dr. S.N. Medical College General Surgery Department in Jodhpur, India, operated on 100 patients. The sample was divided into two 50-person groups. Group A includes emergency surgery patients, while Group B includes elective surgery patients. The samples were aseptically collected and processed according to microbiological methods. Data were processed with IBM SPSS Statistics for Windows, version 20 (released 2011; IBM Corp., Armonk, New York, United States).

Results

Out of a sample size of 100 patients, 17 individuals experienced SSIs. SSI incidence was 16.66% in male patients and 18.18% in female patients. In addition, the rate of SSIs was 26% in the emergency group and 8% in the planned group. The association was stronger among elderly individuals, diabetics (33.33% in Group A and 12.5% in Group B), and anemics with a history of smoking. The association was higher in those who underwent surgery for more than 60 minutes (34.37% in Group A and 18.8% in Group B). The incidence of SSIs was higher in emergency cases compared to elective surgeries, with rates of 26% and 8%, respectively, but was statistically insignificant. The infection rate in clean cases during planned surgery was 3.70%, while clean contaminated cases during planned surgery had a wound infection rate of approximately 13.04%. In emergency surgery, no clean case was operated on, but the SSI rate in the emergency group was 9.09%, 22.22%, and 47.36% in the clean-contaminated, contaminated, and dirty cases, respectively. In Group A, *Escherichia coli* was the predominant organism found in SSI wounds, while in Group B, *Staphylococcus aureus* was the predominant organism, accounting for 46.15% and 50% of infections, respectively. Amikacin and metronidazole exhibited the highest efficacy against *E. coli*, with amikacin demonstrating the highest sensitivity.

Conclusion

SSIs are more common in emergencies than planned procedures. Age, gender, diabetes, hypertension, smoking, and prolonged surgery are risk factors for SSIs. Effective antibiotic policy and infection control can greatly prevent SSIs.

## Introduction

The incidence of surgical site infections (SSIs) in hospitalized patients is estimated to be around 2%. However, this number may be lower than the actual rate due to incomplete postoperative discharge data [1,2]. Additional data suggest that SSIs occur at a rate ranging from 3% to 20% during specific procedures, with a potentially higher incidence among patients considered high risk. SSIs result in substantial morbidity and long-term disabilities due to impaired wound healing and extensive tissue damage. SSIs lead to substantial morbidity and persistent disabilities, resulting in a considerable economic burden and increased healthcare costs. A study found that the cost per patient increased by €814 to €6,626 in the United Kingdom, while in the United States, the estimated cost increased by $1.8 billion annually [3,4]. Contrary to what some surgeons think and believe, an SSI is not a small illness with a benign course. An SSI is a postoperative infection that takes place within the surgical site.

The Centers for Disease Control and Prevention (CDC) in the United States defines an SSI as the occurrence of inflammatory signs or pus discharge within 30 days of a primarily closed surgical incision. The CDC categorizes procedures into four classes (Classes I, II, III, and IV) based on the likelihood of contamination during the operation [5,6,7]. The incidence of SSIs is influenced by the type of surgical procedure and the clinical characteristics of patients undergoing surgery. An SSI is the result of interactions among bacterial inoculation, bacterial virulence, microenvironment of the surgical site, and host defense mechanisms. During the surgical procedure, microorganisms are introduced into the incision site. Pathogenic microorganisms can be acquired from various external sources during surgery, including surgical equipment, implants, gloves, air in the operating room, and medications administered during the procedure [8]. Most of these microorganisms are endogenous flora derived from the patient.

## Materials and methods

Patient data, study population, and sampling technique

The study was conducted at the Department of General Surgery of Dr. S.N. Medical College in Jodhpur, Rajasthan, India, from January 2017 to December 2017. All cases admitted to the surgical wards (including both elective and emergency surgery) during the study period and those who met the criteria for inclusion were included in the study according to the convenience sampling technique. The study involved 100 patients and the sample was stratified into two groups of 50 individuals each. Group A (emergency group) consists of patients requiring emergency surgery due to conditions, such as intestinal obstruction, penetrating abdominal injury, ruptured appendix, gall bladder or intestinal perforation, blunt trauma to the abdomen from a road traffic accident, splenic injury, pyoperitoneum, or obstructed hernia. Group B (planned group) comprises patients undergoing elective surgeries, such as inguinal hernia repair, mastectomy for breast cancer, cholecystectomy, pseudo-pancreatic cyst, and ventral hernia repair.

Inclusion criteria

Participants were required to be at least 15 years of age, have no previous history of infection at the surgical site, and be with or without comorbidities, such as diabetes, hypertension, anemia, history of smoking, or any other chronic illness.

Exclusion criteria

Late-onset surgical site infection (>30 days), age under 15 years, patients undergoing a repeat surgical procedure, immunocompromised state or taking such immunocompromised drug or any steroid, and those operated on for thoracic surgery, obstetrics and gynecology surgery, orthopedic surgery, and head surgery.

Before surgery, the patients underwent a clinical evaluation to assess the onset, progression, and duration of symptoms, such as abdominal pain, distension, nausea, vomiting, constipation, rectal bleeding, and abdominal lumps. All patients underwent a physical examination to assess signs and vital parameters, such as blood pressure and pulse rate. This included an evaluation for abdominal tenderness, guarding, or rigidity. Abdominal X-ray in an upright position to assess the presence of gas under the diaphragm, the presence of air-fluid levels, and foreign bodies. Abdominal ultrasonography was performed to assess the condition of the liver, spleen, peritoneal fluid level, nature of fluid (hemoperitoneum/pyoperitoneum), status of the appendix, intestinal obstruction, dilated bowel loops, abdominal lump, and other related factors.

Following surgeries, the patients were assessed for signs of SSIs, such as erythema, swelling, discharge, tenderness, and increased temperature at the incision site. All the patients underwent postoperative assessment for systemic symptoms, such as fever, nausea, vomiting, and purulent discharge, and were subsequently referred for culture sensitivity analysis. Sick patients underwent complete blood counts and blood culture tests. The wounds were dressed periodically using povidone-iodine and hydrogen peroxide solution (H_2_O_2_), and the administration of antibiotics was based on the results of culture sensitivity reports.

Statistical analysis

The chi-square test was used to test the strength of association for categorical variables. Taking the confidence interval of 95%, the statistical significance was defined as a p-value of less than 0.05. The data were tabulated on a spreadsheet and analyzed using IBM SPSS Statistics for Windows, version 20 (released 2011; IBM Corp., Armonk, New York, United States).

Ethical approval

The research was undertaken after obtaining approval from the Institutional Ethics Committee of Dr. S.N. Medical College (approval number: Dr SN MC/IHECMED/MC/JU/14.03.2018/801).

## Results

The total male patients were 42 and 36 and the female patients were eight and 14 in the emergency and planned groups, respectively (p-value < 0.01). The rate of SSIs was higher in the male patients than the female patients in emergency surgery at 28.57% vs. 12.5%, respectively (Table 1; p-value < 0.119). The rate of SSIs was higher in the female than male patients in planned surgery, i.e., 21.43% vs. 2.78%, respectively (Table 1). The overall incidence of SSIs in the male patients was 16.66% and 18.18% in the female patients, while the overall rate of SSIs in the emergency and planned groups was 26% and 8%, respectively.

**Table 1:** Demographic distribution according to wound infection SSI: surgical site infections; No.: number of patients

Age (years)		No. of patients				P-value
		Emergency group	SSIs	Planned group	SSIs	0.010
	15-30	28 (56%)	4 (14.29%)	16 (32%)	2 (12.50%)	
	31-45	14 (28%)	6 (42.46%)	7 (14%)	0 (0.00%)	
	46-60	6 (12%)	2 (33.33%)	16 (32%)	1 (6.25%)	
	≥60	2 (4%)	1 (50%)	11 (22%)	1 (9.09%)	
	Total	50	13 (26%)	50	4 (8%)	
Sex	Male	42 (84%)	12 (28.57%)	36 (72%)	1 (2.78%)	0.119
	Female	8 (16%)	1 (12.5%)	14 (28%)	3 (21.43%)	
	Total	50	13 (26%)	50	4 (8%)	

**Figure 1:**
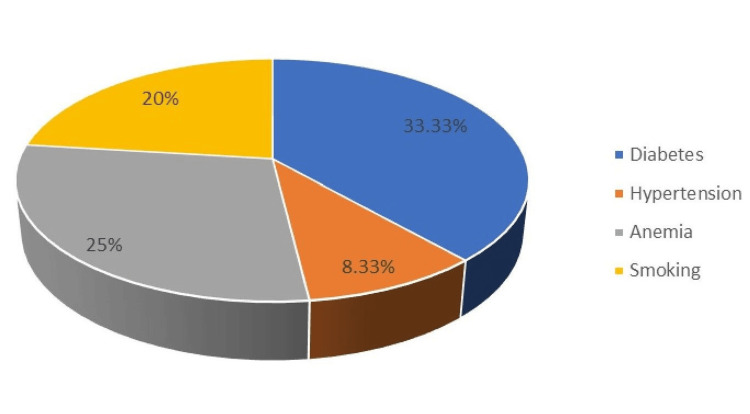
Percentage of SSIs in the emergency group SSI: surgical site infections

**Figure 2:**
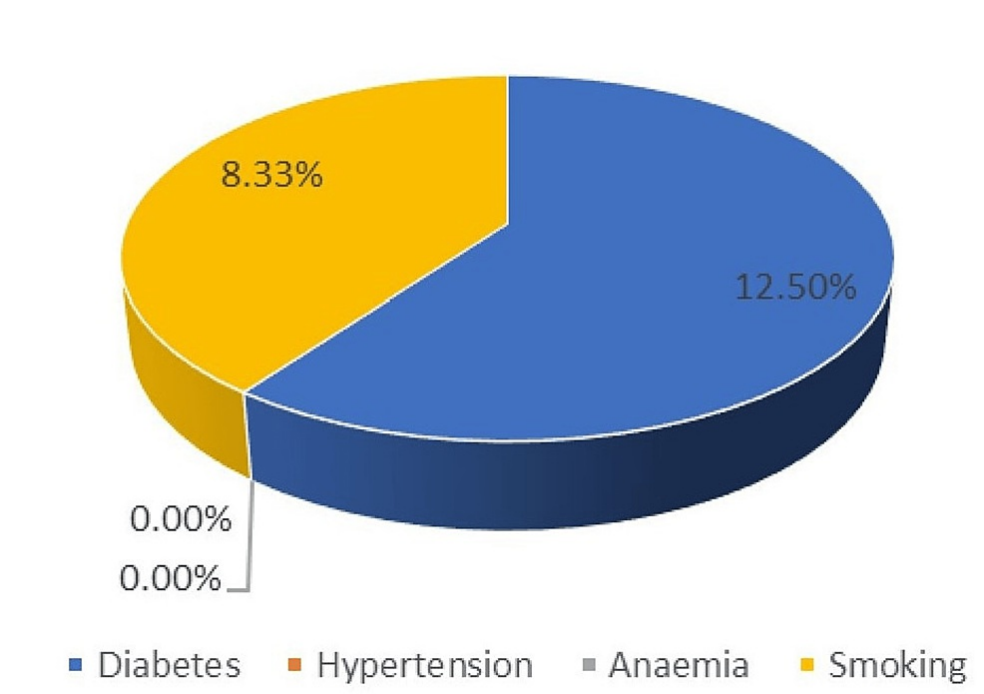
Percentage of SSIs in the planned group SSI: surgical site infections

As shown in Table 2, the incidence of SSIs in patients with diabetes was two out of six in Group A and one out of eight in Group B. In Group A, the incidence of SSIs was 8.33% (one out of 12) among patients with hypertension and 25% (two out of eight) among patients with anemia (Table 2, Figures 1, 2). None of the patients in Group B with hypertension developed SSIs. The incidence of SSIs was 20% and 8.33% among individuals with a history of smoking in the two groups.

**Table 2:** Distribution of SSIs as per comorbidities and smoking status SSI: surgical site infections

Comorbidities	SSIs (%)/no. of patients (Group A)	SSIs (%)/no. of patients (Group B))
Diabetes	2 (33.33%)/6	1 (12.5%)/8
Hypertension	1 (8.33%)/12	0 (0%)/10
Anemia	2 (25%)/8	0 (0%)/1
H/O smoking	4 (20%)/20	1 (8.33%)/12

**Figure 3:**
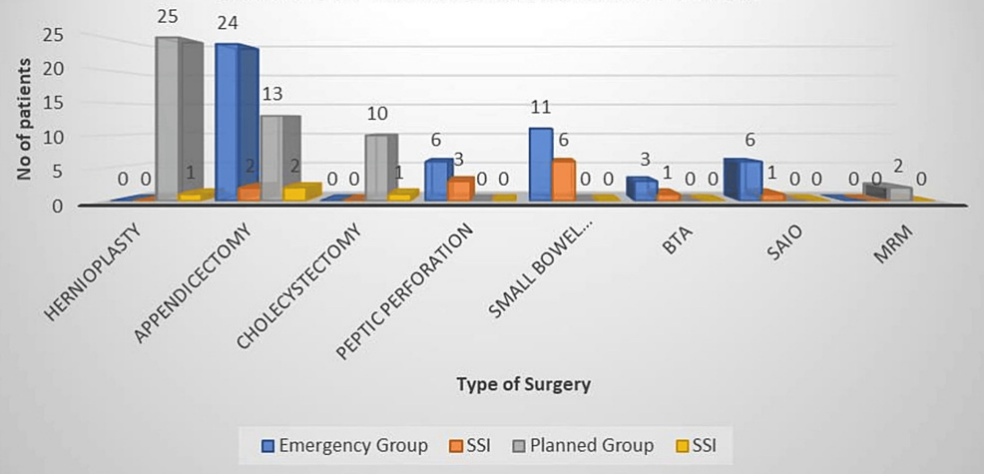
Distribution of infections according to the type of surgery BTA: blunt trauma abdomen; SAIO: sub-acute intestinal obstruction; MRM: modified radical mastectomy Image created with Microsoft Excel

In emergency surgery, according to Table 3 and Figure 3, the risk of SSIs was 8.33% in appendicectomies but was higher in the planned group (15.38%). In cases of peptic perforation, 50% developed SSIs and were operated on only in emergencies. Small intestinal perforations with peritonitis had the maximum SSI risk, accounting for 54.54%. In other cases, such as blunt trauma abdomen, subacute subacute intestinal obstruction, and modified radical mastectomies, the SSI rate was 33.33%, 16.66%, and 0%, respectively. The overall risk of SSI was 26% and 8% in the emergency and planned groups, respectively.

**Table 3:** Distribution of infections according to the type of surgery SSI: surgical site infections; SAIO: sub-acute intestinal obstruction; MRM: modified radical mastectomy

Type of surgery	Emergency group	SSIs	Planned group	SSIs
Hernioplasty	0	0 (.00%)	25	1 (4%)
Appendicectomy	24	2 (8.33%)	13	2 (15.38%)
Cholecystectomy	0		10	1 (10%)
Peptic perforation	6	3 (50%)	0	0
Intestinal perforation peritonitis (ileal and jejunal perforation repair)	11	6 (54.54%)	0	0
Blunt trauma abdomen (laparotomy)	3	1 (33.33%)	0	0
SAIO (laparotomy with adhesiolysis)	6	1 (16.66%)	0	0
MRM	0	0	2	0
Total	50	13 (26%)	50	4 (8%)

In the emergency group, there were no cases that underwent clean surgery. There were two cases of SSIs out of 22 cases of clean-contaminated surgery, two cases of SSIs out of nine cases of contaminated surgery, and nine cases of SSIs out of 19 cases of dirty surgery in the emergency group, as shown in Table 4. In the planned group, no cases were operated on in contaminated or dirty surgery, but one case of SSIs out of 27 was found in clean surgery and three cases of SSIs out of 23 were found in clean-contaminated surgery. The overall infection rate was 17%, with a higher incidence of SSIs observed in the emergency group (26%, p-value = 0.104) compared to the planned group (8%, p-value = 0.264). The results were not statistically significant in either groups, as shown in Table 4.

**Table 4:** Distribution of SSIs in different classes of wounds in emergency vs. planned surgeries SSI: surgical site infections

Group	Emergency		Planned	
	SSIs/total	%	SSIs/total	%
Clean	0	0	1 / 27	3.70
Clean contaminated	2/22	9.09	3/ 23	13.04
Contaminated	2/9	22.22	0	0.00
Dirty	9/19	47.36	0	0.00
Total	13/50	26	4/50	8
P-value	0.104		0.264	

The incidence of SSIs was found to be 11.11% in emergency surgeries lasting less than 60 minutes and 34.37% in those lasting more than 60 minutes. In the planned surgeries, no case of SSIs was observed in surgeries lasting 60 minutes or less, whereas 18.18% of cases developed SSIs in surgeries lasting more than 60 minutes, as per Table 5, with a p-value of 0.087 and 0.319 in cases operated less than and more than 60 minutes, respectively.

**Table 5:** Distribution of infections according to the duration of surgery SSI: surgical site infection

Group	Surgery	<60 minutes		≥60 minutes	
		Cases	SSI	Cases	SSI
Emergency	Clean	0	0	0	0
	Clean contaminated	14	0	8	0
	Contaminated	2	0	7	2
	Dirty	2	2	17	9
	Total	18	2 (11.11%)	32	11 (34.37%)
Planned	Clean	20	0	7	1
	Clean contaminated	8	0	15	3
	Contaminated	0	0	0	0
	Dirty	0	0	0	0
	Total	28	0	22	4 (18.18%)
	P-value	0.087		0.319	

Table 6 indicates that *Escherichia coli*, *Staphylococcus aureus*, and *Pseudomonas* were the most prevalent organisms cultured from postoperative wounds in the emergency group, accounting for 46.15%, 30.77%, and 7.69% of cases, respectively. The study found that after emergency surgery, tachycardia was present in 40% of the patients, fever in 20%, pain and redness or edema in 12%, swelling in 8%, and discharge in 34%. In the planned group, *S. aureus* was the predominant organism at 50%, followed by *Citrobacter* at 25%. Gram-negative bacteria accounted for 57% of the isolates, while gram-positive bacteria accounted for 43% of the isolates. During scheduled surgical procedures, 8% of the patients experienced tachycardia, 4% experienced redness or edema, and 6% experienced a combination of fever, pain, swelling, and discharge (Table 6).

**Table 6:** Distribution of organisms cultured from the infected post-operative wound and the clinical examination for SSI examination in both groups N: number of patients; SSI: surgical site infection

		Emergency group		Planned group	
		N	%	N	%
Organism	*Escherichia* *coli*	6	46.15	0	0.00
	Staphylococcus aureus	4	30.77	2	50.00
	Pseudomonas	1	7.69	0	0.00
	Citrobacter	0	0.00	1	25.00
	Total	11	84.62	3	75.00
Examination (general/local)	Tachycardia	20	40.00	4	8.00
	Fever	10	20.00	3	6.00
	Pain	6	12.00	3	6.00
	Redness/edema	6	12.00	2	4.00
	Swelling	4	8.00	3	6.00
	Discharge	17	34.00	3	6.00

Amikacin and metronidazole were the two most frequently effective antibiotics against *E. coli*, with amikacin being the most commonly sensitive (Table 7). Azithromycin and amikacin were the top two sensitive antibiotics for *S. aureus*.

**Table 7:** Antibiotic sensitivity on different organisms

Sensitivity	Organism			
	Escherichia coli	Staphylococcus aureus	Pseudomonas	Citrobacter
Amikacin	5	3	0	1
Metronidazole	3	1	0	0
Azithromycin	1	4	0	0
Linezolid	0	2	0	0
Ceftriaxone	2	0	0	0
Meropenem	1	0	0	1
Imepenem	0	0	0	0
Moxifloxacin	0	0	1	0
Tobramycin	0	0	1	0
Ciprofloxacin	0	1	0	0
Tetracyclin	0	1	0	0

## Discussion

SSIs are still a significant issue in all surgical specialties in hospital settings, despite numerous improvements in asepsis, antimicrobial medications, sterilization, and surgical methods. The rising cost, morbidity, and death associated with surgical procedures are caused by these infections. The cost of hospitalization nearly doubles for any given type of operation when a wound infection develops [5-7]. In addition to driving up hospital expenses, SSIs can cause patients to acquire antimicrobial resistance, which can then spread to other patients in the neighborhood and have an impact on primary care. This study aims to assess the occurrence of SSIs in our setting, identify the types of bacteria causing SSIs, analyze their antibiotic resistance patterns, and examine their relationship with associated risk factors.

In planned surgery in the present study (Table 4), 3.70% (one out of 27) of clean cases and 13.04% (three out of 23) of clean-contaminated cases developed SSIs. In emergency surgery, no SSI was observed, because no clean case was operated on. However, in the emergency group, 9.09% (two out of 22) clean-contaminated, 22.22% (two out of nine) contaminated, and 47.36% (nine out of 19) dirty cases developed SSIs (Table 4). In the emergency group, the rate of SSIs was 26% (13 out of 50), as compared to 8% (four out of 50) infections in the planned group. However, the findings were not statistically significant (p-value was 0.109 and 0.265 in the emergency and planned groups, respectively). The overall infection rate was 17% in the present study, which is highly connected with infection rates ranging from 6.09% to 38.7% in many previous studies [7-9]. Other developed countries have reported lower infection rates ranging from 2.8% to 19.4% [10-17].

SSIs are the most common healthcare-associated infection that significantly contributes to morbidity and mortality among individuals undergoing surgical procedures [9,18]. The incidence of SSIs exhibits variability across different hospitals. SSIs range from 2.5% to 41.9% [19].

Considering demographics, including age and gender, the emergency group had the highest number of patients aged 15-30 years, whereas the planned group had the highest number of patients aged 15-30 and 45-60 years. The rate of SSIs was high among males in the emergency group, while it was high among females in the planned group and among females overall accounting for 18.88% versus 16.66% in males. However, these demographic findings were statistically insignificant (Table 1). The rate of SSIs was more in females than male patients in planned surgery (21% vs. 2.7%, respectively). Many studies showed SSI proportion among males as 29% and females as 10% [20,21]. Khan et al. reported a marginal preponderance of females (14.79%) over males (14.35%) with SSIs [22].

Previous studies conducted in India have reported SSI rates as high as 49.50% [8]. The American College of Surgeons National Surgical Quality Improvement Program (ACS-NSQIP) dataset was used in a 2005-2008 cross-sectional study of 34,426 cases. The cases included 49.7% clean, 35.0% clean/contaminated, 8.56% contaminated, and 6.7% dirty. Clean, clean/contaminated, contaminated, and unclean wounds had 1.76%, 3.94%, 4.75%, and 5.16% superficial SSI rates, respectively. Deep incisional infections were 0.54-2.1%. Organ/space infections were 0.28-4.54%. The present study also revealed the association of increased rate of SSIs with operative time, diabetes, smoking, anemia, and number of blood transfusion (Tables 2, 5 and Figures 1, 2). Ortega et al. estimated SSI rates as 1-5%, 3-11%, and 10-17% in clean, clean-contaminated, and contaminated cases, respectively [23].

Hernioplasty was the clean case, which was operated only in the planned group, and out of 25, only one had an SSI, i.e., in 4% of cases. Appendicectomy was the most common surgery in the emergency group, but the rate of SSIs was observed only in 8.33% of cases. The highest rate of SSIs was observed in cases of intestinal perforation, which may be because of the presence of bacterial flora in the intestinal contents (Table 3 and Figure 3). All peptic perforations were operated on in the emergency, with a 50% risk of SSIs. Patients with intestinal or peptic perforation were stabilized in the intensive care unit (ICU) for hemodynamic stability before surgery and moved to the ICU afterward. The overall rate of SSIs was high in the emergency group, which is linked to the type of surgical pathology, especially the one that is associated with more infected contents. Razavi et al. reported in their study the incidence of SSIs in appendectomy (10-20%), hydatid cysts (2-5%), acute and chronic cholecystectomy (10%), laparoscopic cholecystectomy (2-5%), and intestinal surgery (20%) [24].

In our study emergency group, 11.11% of cases developed SSIs in surgeries of 60 min duration or less, while 34.37% of cases developed SSIs in surgeries of more than 60 min duration. In the planned group, no cases developed SSIs in surgeries of 60 min duration or less, while 18.18% of cases developed SSIs in surgeries of more than 60 min duration (Table 5). Our study's findings are consistent with those of Cheng et al. [25]. The study found a higher association with statistically significant findings between longer operative times and SSIs in the pooled analyses. The probability of SSIs increased almost twofold across various time intervals. The study found that the likelihood of a certain outcome increased by 13% for every 15 minutes, 17% for every 30 minutes, and 37% for every 60 minutes of surgery [25]. Patients with SSIs had a significantly longer mean operative time compared to patients without SSIs, with an approximate difference of 30 minutes.

Organisms cultured from wound swabs or collections were predominantly gram-negative (57%) as compared to gram-positive (43%). Among the organisms cultured from the postoperative wound, *E. coli* was the most common organism (46.15%), followed by *S. aureus* (30.77%) and *Pseudomonas* (7.69%) in emergency surgeries. In the planned group, the most common organism is *S. aureus* (50%), and the second most common organism is *Citrobacter *(25%) (Table 6). *S. aureus* continues to be the pathogen most frequently found in SSIs worldwide [26]. Following it are *Enterococcus* sp., *E. coli*, *P. aeruginosa*, and *Enterobacter* sp. Other pathogens involved in the infection are *Proteus mirabilis*, *Klebsiella pneumoniae*, *Candida albicans*, and different *Streptococcus* species [26]. The National Nosocomial Infections Surveillance (NNIS) system data show that there has been no notable alteration in the distribution of pathogens found in SSIs in the last 10 years [27]. Common pathogens include *S. aureus*, coagulase-negative staphylococci, *Enterococcus* spp., and *E. coli*. Antimicrobial-resistant pathogens like methicillin-resistant *S. aureus* (MRSA) and *Candida albicans* are becoming more common. This means that these pathogens are causing more SSIs [28].

A significant relationship was observed between the rate of infection and variables, such as wound type, surgery duration, and whether the surgery was emergency or planned. The most frequently identified organisms in patients with SSIs were gram-negative bacteria, specifically *E. coli* that produce extended-spectrum β-lactamase. This finding contradicts previous studies that reported a higher prevalence of gram-positive bacteria, specifically *S. aureus* and coagulase-negative staphylococci [29].

In this study, out of 14 wound infections, eight were caused by a gram-negative organism and six was caused by a gram-positive organism. Mangram et al. [26] found that gram-negative bacilli (e.g., *E. coli*), gram-positive organisms (e.g., *Enterococci*), and sometimes anaerobes (e.g., *Bacteroides fragilis*) are the most common pathogens found in gastrointestinal SSIs. In addition to endogenous sources, external factors, such as the surgical team, the operating room environment (including air), and all tools, instruments, and supplies introduced into the sterile field during an operation contribute to SSIs. The exogenous flora primarily comprises aerobes, specifically gram-positive bacteria like *Staphylococci* and *Streptococci*. Fungal infections are rarely implicated in the etiology of SSIs, which can arise from endogenous or exogenous sources. Amrutham et al. discovered that *S. aureus* was the predominant organism (53.33%) isolated from surgical sites [30]. The subsequent bacterial isolates were as follows: *P. aeruginosa* (35.55%), *K. pneumonia* (26.66%), *E. coli* (17.77%), *Proteus *(13.33%), *Staphylococcus epidermidis* (11.11%), *Citrobacter* (8.88%), and *Enterobacter* (11.11%). Most isolates showed resistance to multiple drugs [30]. Amikacin was the most common sensitive antibiotic for *E. coli*, followed by metronidazole (Table 7). Azithromycin is the most common sensitive antibiotic for *S. aureus*, followed by amikacin.

The current study is subject to certain limitations, namely, the relatively small sample size and the inclusion of a single centre. Increased patient sample size and additional studies conducted across multiple centers, along with various systematic reviews or meta-analyses, could potentially provide further validation for the findings of the current study.

## Conclusions

If comorbidities, such as diabetes, hypertension, and anemia, are corrected or well controlled, the possibility of SSIs can be minimized to a certain extent. In the present study, the rate of SSIs was observed to be high in cases having such diseases in both emergency and elective surgeries. Considering contaminated and dirty surgeries, the duration of the surgery was also an important factor in the present study regarding SSIs. A surgical duration of more than 60 minutes is associated with a high rate of SSIs in both emergency and elective surgeries, although the findings were not significant. *E. coli*, *S. aureus*, and *Pseudomonas* were the most prevalent organisms cultured from postoperative wounds in the emergency group, while in elective surgery, *S. aureus* was the predominant organism, followed by *Citrobacter*. Antibiotic prophylaxis and post-operative antibiotics based on culture and sensitivity can help reduce morbidity and mortality in cases that are associated with SSIs.
